# Middermal Elastolysis: Dermal Fibroblasts Cooperate with Inflammatory Cells to the Elastolytic Disorder

**DOI:** 10.1155/2017/9524594

**Published:** 2017-09-17

**Authors:** Giovanna De Cunto, Arianna Lamberti, Maria Margherita de Santi, Clelia Miracco, Michele Fimiani, Giuseppe Lungarella, Eleonora Cavarra

**Affiliations:** ^1^Department of Molecular and Developmental Medicine, Section of General Pathology, University of Siena, Via Aldo Moro 6, 53100 Siena, Italy; ^2^Department of Medicine, Surgery, and Neurosciences, Unit of Dermatology, University of Siena, Viale Bracci, 53100 Siena, Italy; ^3^Unit of Pathological Anatomy, AOU Siena, Viale Bracci, 53100 Siena, Italy; ^4^Department of Medicine, Surgery, and Neurosciences, Unit of Pathological Anatomy, University of Siena, Viale Bracci, 53100 Siena, Italy

## Abstract

Little is known about the cause and pathophysiology of middermal elastolysis (MDE). In this condition, variable inflammatory infiltrate may be present or not together with loss of elastic fibres in the middermis that spares both papillary and lower reticular dermis. MDE may be a consequence of abnormal extracellular matrix degradation related to an imbalance between elastolytic enzymes released from inflammatory and resident cells and their naturally occurring inhibitors. However, the cause of this imbalance is still an object of investigation. In order to shed light on the role of fibroblasts in MDE, we used fibroblast cultures from MDE and control subjects to evaluate matrix metalloproteinases (MMPs) and their major inhibitor TIMP-1, which in combination with neutrophil or macrophage proteases released in inflamed areas may influence the elastolytic burden. We demonstrate that fibroblasts derived from MDE produce *in vitro* low levels of TIMP-1, the major inhibitor of MMPs. Elevated levels of MMP-2, MMP-14, and TIMP-2 capable to activate in a cooperative manner pro-MMP-2 are present in MDE tissue samples. Additionally, significant reaction for MMP-1 is present in the same MDE areas. These data all together suggest that ECM changes in MDE are due to cooperation of different cell populations (i.e., inflammatory cells and fibroblasts).

## 1. Introduction

Middermal elastolysis (MDE) is a rare disease of dermal connective tissue, described for the first time by Shelley and Wood in 1977, as “wrinkles due to idiopathic loss of elastic tissue of the middermis“[1]. Since its first description, approximately 80 cases have been reported in literature; however, it is believed that its true incidence is underestimated. It mostly affects Caucasian young or middle-aged women (30–50 years), rarely men (usually interested in more advanced age); familiar forms are not described [2]. The disorder exclusively involves the skin, and no extracutaneous manifestations have been documented [3].

Clinically, it may appear with patches of fine wrinkling, perifollicular papular protrusions (“peau d'orange” appearance), and inflammatory skin changes, such as reticular erythema. There are conflicting views on the nature of the different clinical variants that for many authors may represent different stages of the same disease. Thus, the histopathological changes reported above may represent in ascending order the different chronological aspects of the same disease. Erythema can appear at first, but it disappears afterwards; however, in some patients, erythematous lesion develops and persists hereafter as reticulate erythema. The true course of the disease has not yet been exactly established because only isolated cases of this rare condition reach a dermatologist's observation. At light microscopy, MDE can be appreciated as selective loss of elastic fibres in the middermis sparing both papillary dermis and lower reticular dermis. A variable inflammatory infiltrate may be present or not according to the age of the lesion. At the present time, the aetiology is unknown and the role of sun exposure is still an object of discussion [3–5].

A role for elastolytic activities has been put forward by some authors [3–5], who consider the disease as a possible consequence of an abnormal extracellular matrix degradation, caused by a defect of elastin maturation because of a decrease of lysyl oxidase-like 2 (LOX2) activity [6] and/or by an increase of serine- or matrix metalloprotease activities (MMPs). This may result in an imbalance between these enzymes and their naturally occurring inhibitors [7, 8].

In order to shed light on the role of fibroblasts in MDE, we used *in vitro* cell cultures of fibroblast from MDE and healthy control subjects. By using different methodological approaches, we evaluated fibroblast MMPs and their major inhibitor TIMP-1, which by their own or in combination with neutrophil or macrophage serine proteases may increase the elastolytic burden in the middermis. The results we obtained *in vitro* were together with those obtained in MDE tissues in which inflammatory cells are present or not.

## 2. Materials and Methods

### 2.1. Light and Electron Microscopy

Specimens for this investigation were derived from patients whose clinical history is summarized below. For light microscopy, cutaneous tissue was fixed in 10% buffered formalin and embedded in paraffin wax. Tissue slides were further processed and stained with haematoxylin and eosin (H&E) and Giemsa-Orcein.

For transmission electron microscopy (TEM), the tissue was fixed in 2.5% glutaraldehyde in 0.1% cacodylate buffer, washed in the same buffer, postfixed in 1% osmium tetroxide, dehydrated in ethanol, and embedded in Epon/Araldite. Ultrathin sections were stained with uranyl acetate and lead citrate and observed in a Philips EM 400.

### 2.2. Isolation and Culture of Human Fibroblasts from Skin Biopsy

Primary fibroblast cultures from the skin specimens obtained from MDE patients and control subjects were isolated by explant technique from de-epidermized dermal biopsies.

Tissue specimens were washed in D-MEM with antibiotics, finely minced and allowed to adhere to plastic flasks. Dermal pieces were removed from the culture dish when adherent cells were visible on the plastic surface surrounding tissue fragments. The cultures were grown in D-MEM supplemented with antibiotics, L-glutamine, and 10% FCS at 37°C in a humidified atmosphere containing 5% CO_2_. The medium was changed every other day up. At passage 4, dermal fibroblasts were characterized for standard cell surface markers, namely, Vimentin and CD90 (Thy), and cell growth was assessed by cell count. Subconfluent cultures were washed three times with D-MEM, incubated for 24 hours in serum-free medium. The supernatants were collected in test tubes and kept at −80°C until zymographic analysis was performed. Adherent cells were treated with TRI Reagent (AMBION) in order to recover DNA after cell lysis.

### 2.3. ELISA Assay

The quantitative determination of TIMP-1 was carried out by using samples from three different culture media of fibroblasts derived from each subjects. Determination was carried out by using “Invitrogen Hu TIMP-1 ELISA kit,” according to the manufacturer's instructions.

All the samples were diluted 1 : 20 with standard diluent buffer. Measurements were performed on triplicate samples (standards and tissue samples) by using a microtiter plate reader (Victor3 1420 MultiLabel Counter—PerkinElmer; equipped with software Wallac 1420 Station) at 450 nm.

### 2.4. Zymography

The gelatinolytic activities related to MMP-2 and MMP-9 were visualised by gelatin zymography in the supernatant from three different culture media of fibroblasts derived from each subjects. The molecular weight markers and samples were electrophoresed under nonreducing conditions by SDS-PAGE in 10% polyacrylamide gels copolymerized with 1% gelatin (Biorad). Before loading, the amount of protein in samples was normalized by DNA content determined spectrophotometrically at A_260_ after RNA extraction with TRI Reagent (Ambion). All samples were diluted 1 : 1with “Zymogram sample buffer” (Biorad).

After electrophoresis, gels were washed vigorously twice for 15 min in 2.5% Triton X-100 to remove SDS, then incubated in 50 mM Tris/HCl, pH 7.5, 5 mM CaCl2, at 37°C overnight. Gels were stained with 0.5% Coomassie blue G250 for 3 hours. After destaining, the MMP activity was detected as clear bands against the blue background.

The same protocol was carried out to reveal an activity likely related to MMP-3 on 12% polyacrylamide gels copolymerized with 1% casein (Biorad).

An additional zymographic analysis was carried out by loading the active human MMP-2 (Abcam, ab81550, active human MMP-2 full length protein) together with our samples and molecular weight standard. This was done in order to confirm that the most relevant active band we observed in gelatin gel is related to MMP-2.

### 2.5. Skin Samples

For this study, different samples of skin taken from two female patients with typical features of MDE were used. In particular, tissues in which MDE changes are associated or not with inflammatory reaction were processed and utilized for preparing primary cell cultures. Control samples were taken from healthy donors who gave informed consensus for biopsies.

Briefly, patient number 1 is a 56-year-old Caucasian woman who presented with one-year history of a persistent reticulate slightly itching erythema, on upper chest, shoulders, and proximal upper limbs. Skin examination also revealed, on the back, many round areas where the skin appeared “orange peel.” No other cutaneous or systemic diseases were found in the medical history. In addition, a photo-test was performed to exclude any potential photo-induced dermatoses. Skin biopsies for light and electron microscopy were used for diagnosis and to exclude other elastolytic disorders of the skin [9].

Patient number 2 is a 40-year-old Caucasian woman who came to our clinic with a three-year history of asymptomatic, well-demarcated, skin-colored finely wrinkled patches, ranging from 1 to 4 cm in diameter, on the trunk and upper limbs, and a reticulate erythema on the chest; furthermore, perifollicular papules were noticed on the back. Over the past six months, she had noticed ring-shaped erythematous patches, sometimes confluent to delineate strange polygonal figures, located distally on the thighs and forearms; some of those had become increasingly wrinkled. Medical history revealed a mild form of fibromyalgia, and laboratory tests were normal. Two biopsy specimens were taken from affected areas on the thigh (the most recent lesions) and thorax (the older ones). Tissue samples were processed for light and electron microscopy for diagnostic use and to exclude other elastolytic disorders of the skin.

### 2.6. Immunohistochemical Analysis

Tissue sections (7 *μ*m) from middermal elastolysis or healthy control skins were used for immunohistochemical analysis of MMP-3, MMP-9, MMP-2, MMP-1, TIMP-2, and MT1-MMP (MMP-14).

In this context, we used primary rabbit polyclonal antibodies against human metalloproteinase 3 (MMP-3) (1 : 100; Novus Biological, NB100–91878), metalloproteinase 9 (MMP-9) (1 : 250; Novus Biological, NBP1–57940), metalloproteinase 2 (MMP-2) (1 : 500; Novus Biological, NB200–193), metalloproteinase 14 (MT1-MMP) (1 : 100; Millipore, AB6004), and rabbit polyclonal antibodies against mouse iNOS (1 : 100, Abcam Ltd., Cambridge, UK). Additionally, we used mouse monoclonal antibody to reveal human TIMP-2 (1 : 400; Millipore MAB 3310) and metalloproteinase 1 (MMP-1) (1 : 50; Arigo Biolaboratories, ARG21506).

All the sections were pretreated with 3% hydrogen peroxide for endogenous peroxidase blocking. Antigen retrieval was performed by heating sections in a microwave oven for 20 min in 0.01 M citrate buffer, pH 6.0, and allowing to cool slowly to room temperature.

All the sections were incubated with 3% bovine serum albumin for 30 min at room temperature to block nonspecific antibody binding and then incubated with the respective primary antibodies, overnight at 4°C.

Subsequently, tissue sections were rinsed with PBS, incubated with sheep anti-rabbit IgG (diluted 1 : 200) for 30 min at room temperature followed by incubation with peroxidase-antiperoxidase complex, prepared from rabbit serum. Color development was performed using DAB as chromogen. As negative controls for the immunostaining, primary antibodies were replaced by nonimmunised rabbit serum. The M.O.M. kit was used for immunodetection of mouse monoclonal antibodies against TIMP-2 and TIMP-1 (Transduction Laboratories, Lexington, KY, USA).

### 2.7. Statistical Analysis

Data are expressed as means ± SD. The significance of the differences was calculated using one-way analysis of variance. A *P* value of less than 0.05 was considered significant.

## 3. Results

### 3.1. Microscopy

**Figure 1:**
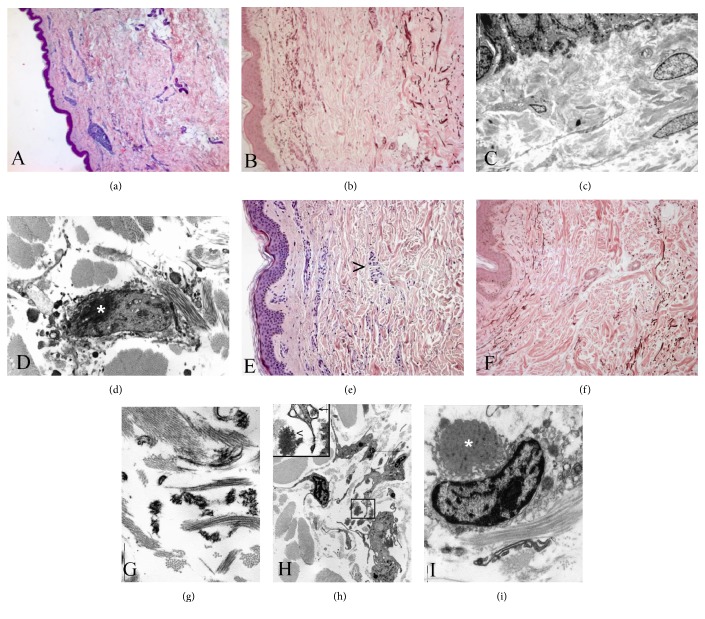
(a–d) Skin biopsy from patient number 1. Inflammatory infiltrates in the upper dermis (a and b) are associated with small areas of elastinolysis in the middermis (b). At TEM examination, there is an almost complete loss of elastic fibres in the middermis. No alterations are seen in the upper dermis (c). *Elastophagocytosis* (white star) by macrophages can be also appreciated (d). (a) H&E stain, ×40; (b) Giemsa-Orcein stain, ×100; (c) and (d) uranyl acetate & lead citrate, original magnification ×13.000. (e–i) Skin biopsy from patient number 2. Large inflammatory infiltrates are present in the middermis (e) where a band-like loss of elastic fibres can be appreciated (f). At TEM examination, a fragmentation of elastic fibres is evident in the middermis (g) ((h), inset arrowhead) where a lot of activated macrophages with an irregular surface are scattered within the extracellular matrix (h). Several macrophages are engaged in *elastophagocytosis* ((i), white star; (h), inset, arrow). (e) H&E stain, ×100; (b) Giemsa-Orcein stain, ×100; (g–i) uranyl acetate & lead citrate, (g) and (i) original magnification ×13.000, (h) original magnification ×7000.

At light microscopy, tissue specimens taken form patient number 1 reveal a focal decrease of elastic fibres in the upper dermis and a band-like loss of elastic fibres in the middermis. A perivascular inflammatory infiltrate is seen around vessels of the upper dermis (Figure 1(a)). In the middermis, some histiocytes are scattered among the collagen bundles (Figures 1(a) and 1(b)) where some patchy areas of elastinolysis are appreciated. At transmission electron microscopy (TEM), no evident changes are seen in the upper dermis (Figure 1(c)), and an almost complete loss of elastic fibres is present in the middermis region (Figure 1(d)). The changes mainly affect the amorphous component of elastic tissue rather than the microfibrillar one. Elastic tissue appears irregularly aggregated, and degenerated elastic fibres are present in phagosomes of macrophages within the extracellular matrix (ECM) (Figures 1(d) and 1(h)). The latter cells with an irregular surface and an active phagocytic activity show the characteristic morphological features of M2-polarized macrophages. These pathological findings are typical changes in MDE.

Haematoxylin and eosin stains of biopsy specimens taken from patient number 2 demonstrated flattened epidermis without alterations, homogeneous papillary dermis, and areas of reticular dermal oedema (Figure 1(e)). In this patient lymphocyte and monocyte, inflammatory infiltrate is particularly evident (arrowhead). In some areas, a significant neutrophil infiltrate is seen. A band-like loss of elastic fibres in the middermis can be appreciated after elastic tissue stain (Figure 1(f)). At TEM examination (Figures 1(g) and 1(h)), the loss of elastic fibres in the middermis is apparent and the *elastophagocytosis* of damaged elastic fibres is also appreciated within macrophages (Figure 1(i)). These features are morphological characteristics of MDE.

### 3.2. Zymographic Analysis

**Figure 2:**
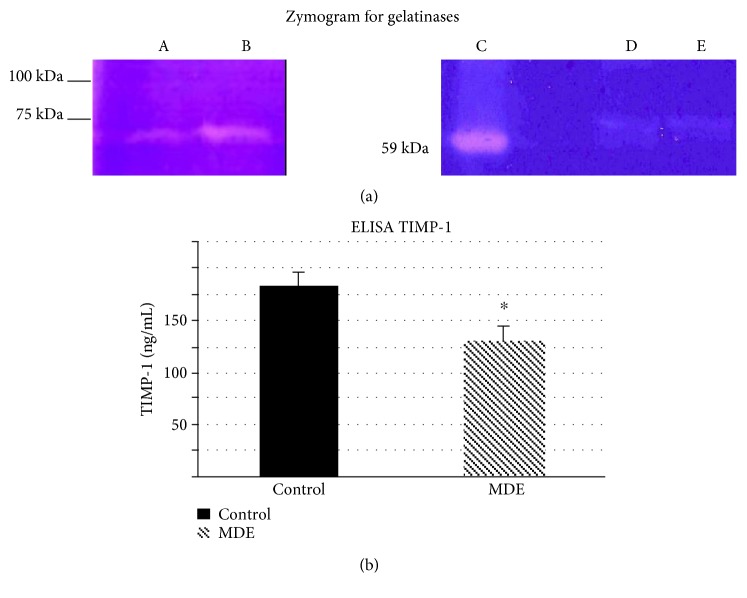
(a) Representative zymogram for gelatinases. (A) supernatant of fibroblast culture from a control subject; (B) supernatant of fibroblast culture from a MDE patient; (C) active form of human MMP-2 (59 kDa form), (D) and (E) are the same samples of (A) and (B), respectively. (b) Quantitative determination of TIMP-1 by ELISA. The values are mean ± SD of triplicate determinations on supernatants from three fibroblast cultures of MDE patients and three controls. ^∗^*p* < 0.05 versus control samples.

In order to identify stromelysins, zymographic analysis was carried out on casein gels. By using this methodology, no appreciable bands either compatible with MMP-3 or MMP-7 molecular weights were revealed in supernatant samples of cultures derived from MDE and healthy areas (data not shown). On the other hand, zymographic analysis on gelatin gels carried out to reveal gelatinases (i.e., MMP-2 and MMP-9) resulted in the appearance of a single 72 kDa band (Figure 2(a)). This band looks more intense in fibroblasts derived from MDE areas (lane b) than those taken form healthy subjects (lane a).

According to molecular weight (~72 kDa), the band found in both MDE patients (lane b) corresponds to the inactive form (zymogen) of human MMP-2, since the active form of human MMP-2 has a relative mobility corresponding to ~59 kDa (lane c) [9].

Representative zymogram of supernatants from fibroblast cell cultures of two MDE patients and control subjects is reported in Figure 2(a), lanes d and e, respectively.

### 3.3. ELISA for TIMP-1

TIMP-1 reduction (of approximately 30%) was observed in MDE fibroblasts (128 ± 18 ng/ml) as compared with that detected in fibroblasts of control samples (183 ± 15 ng/ml) (Figure 2(b)).

### 3.4. Immunohistochemistry

**Figure 3:**
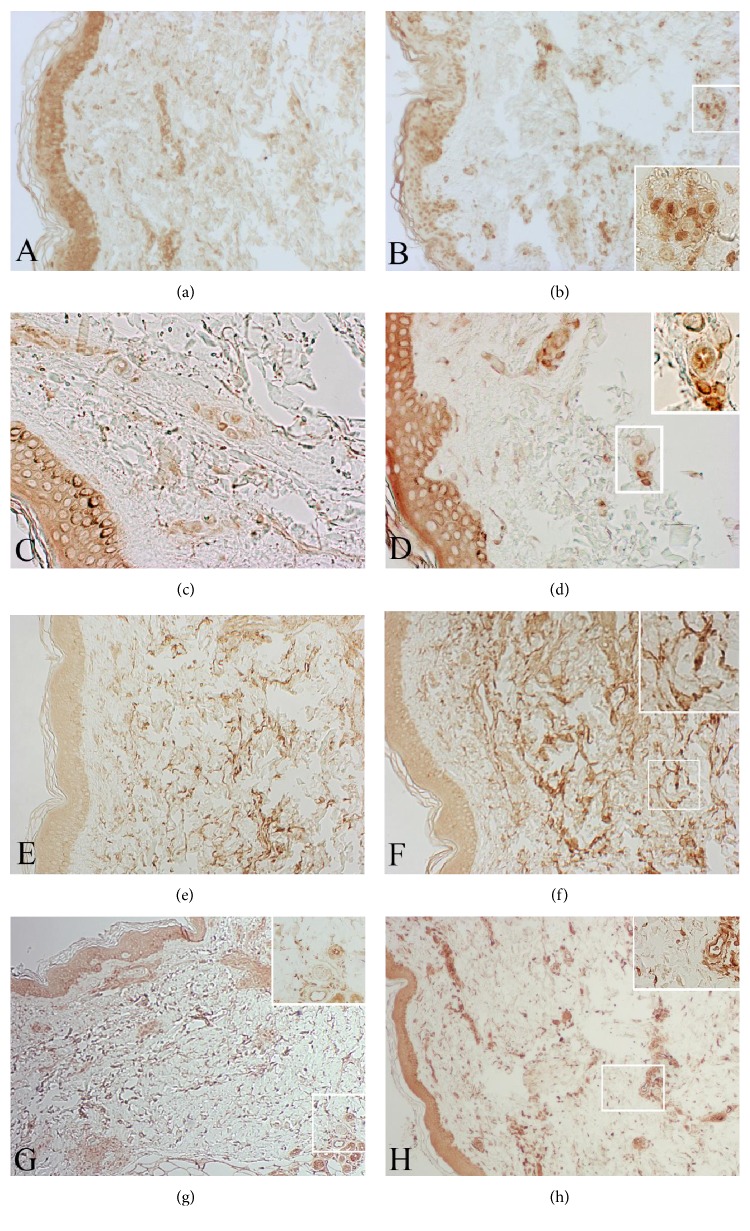
Skin biopsies from MDE patient (b, d, f, and h) and healthy donor (a, c, e, and g). M1 macrophages (iNOS positive) are present in MDE areas together with iNOS negative macrophages (b). No reaction is noticed in the middermis of control patients (a). A weak reaction for MMP-2 (c) and TIMP-2 (e) is detected on cell membranes in the middermis of a control subject. A positive reaction for MMP-2 (d) and TIMP-2 (f) is evident on the cell surface of middermal fibroblasts and inflammatory cells. A diffuse reaction for MMP-14 is localized in large areas of the middermis from MDE patient (h). A mild reaction for MMP-14 is present also on cell membranes in the middermis from a control subject (g). (a-b) Original magnification ×100; (c-d) original magnification ×200; (e-f) original magnification ×100; original magnification ×40.

In order to see whether destructive macrophages are present in MDE together with scavenger macrophages, we performed an immunohistochemical study to detect iNOS, a marker of M1 phenotype. A strong positive staining for iNOS has been observed in several middermis macrophages of patients with MDE (Figure 3(b)). No reaction was noticed in the middermis of control patients (Figure 3(a)). This suggests that destructive M1 macrophages (iNOS positive) together with M2 macrophages (with active phagocytosis) may be at the same time present in the skin of MDE patients.

Evident positive reactions for MMP-2 (Figure 3(d)) and for TIMP-2 (Figure 3(f)) have observed on the cell surface of middermal fibroblasts and inflammatory cells. Only weak reactions for MMP-2 (Figure 3(c)) and TIMP-2 (Figure 3(e)) have been detected on cell membranes in the middermis of control subjects. With regard to MMP-14, a diffuse reaction for this enzyme was localized in large areas of the middermis from MDE patients (Figure 3(h)). A mild immunohistochemical reaction for MMP-14 is detectable also on cell membranes in the middermis from control subjects (Figure 3(g)).

**Figure 4:**
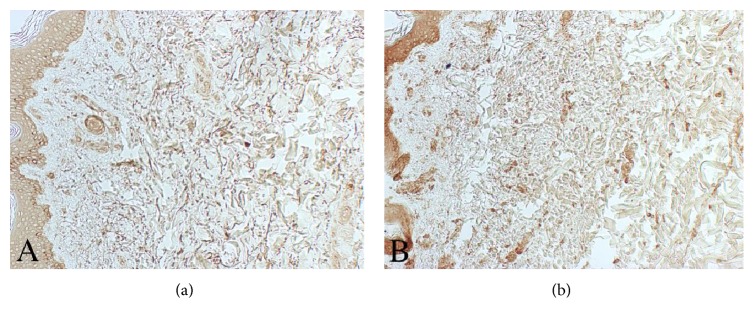
Representative skin samples from control subject (a) and MDE patient (b) after immunoreaction for MMP-1. As can be appreciated, a strong reaction for MMP-1 is found in the upper and middermis of the MDE tissue. Original magnification ×100.

Immunostaining for MMP-3 does not show a positive reaction both in MDE and control subjects (data not shown). On the other hand, a weak staining for MMP-9 is observed on MDE epidermal keratinocytes and histiocytes. A trivial reaction for this enzyme is also present in tissue samples of healthy control subjects (data not shown). Additionally, a strong reaction for MMP-1 can be appreciated in the upper and middermis of MDE tissues by using a monoclonal antibody that recognizes full length MMP-1 (Figure 4).

## 4. Discussion

In this study, we demonstrate that fibroblasts, derived from MDE skins, produce *in vitro* low levels of TIMP-1, the major inhibitor of matrix metalloproteinases and elevated levels of pro-MMP-2. These findings have been confirmed by immunohistochemical analysis carried out on tissues derived from MDE and healthy subjects. Of interest, MMP-14 and TIMP-2 capable to activate on cell surface pro-MMP-2 in a cooperative manner [10] are present in the middermis. In addition, significant amount of MMP-1 is observed in MDE tissue.

The data we reported strongly suggest that middermal elastinolysis is due to cooperation of different cell populations (i.e., inflammatory cells and fibroblasts).

Actually, very little is known on the pathogenic events that lead to MDE [3]. In almost half of the cases reported, sun exposure was implicated and/or the lesions were photo-distributed [2–4]. However, in our patients, there is no histological evidence of chronic sun damage or involvement of chronically sun-exposed sites. Also, the role of inflammation in this pathology is still an object of discussion [3, 9]. The first reports on this condition exclude that inflammation plays a relevant role because no inflammatory infiltrate was found associated with MDE changes [1]. On the contrary, recent observations suggest the possibility of an inflammatory cause in several cases [4, 11–13]. The presence of reticular erythema associated with the classical changes of MDE strongly supports this idea. Additionally, as reported in our paper, inflamed areas are characterised by the presence of activated macrophages with different phenotypes in the middermis. The data we report here suggest that MDE changes may be the result of destructive events due to a cooperation between fibroblasts and inflammatory cells. It is well known that inflammatory cells, namely, neutrophils and macrophages, can secrete destructive enzymes active against many components of ECM and can produce oxygen species capable to activate MMPs [14]. These enzyme activities falling into different classes (serine-, metallo-, and asparticproteases) can degrade ECM components by themselves, or in cooperation. In particular, neutrophil elastase or MMP-12 can promote elastolysis [14–16]. The activity of these enzymes is counteracted by naturally occurring inhibitors such as *α*1-proteinase inhibitor (*α*1-PI) and TIMP-1, respectively [17]. It has been reported that several ECM components are altered in MDE, including collagens [3, 4]. The involvement of different populations of cells in MDE lesions may be of pathogenic importance. In this regard, high levels of MMP-1 may play an additional role that contributes to the various changes that characterize MDE, by promoting proteolysis of ECM components and by recruiting neutrophils [14]. This issue deserves further investigation. As reported in this paper, we demonstrated in supernatants of MDE fibroblast cultures, higher pro-MMP-2, and lower TIMP-1 levels in comparison to those we detected in cultures of control fibroblasts. The low TIMP-1 activity together with high presence of MMP-2 that can promote proteolysis of *α*1-PI (the major inhibitor of serine proteases) may favour and trigger the degradation of elastic tissue by increasing the elastase burden in an inflamed tissue. In addition, active MMP-2 can participate to the development of middermal changes by activating growth factors and chemokines [18, 19]. The presence in MDE areas of molecules (MMP-14 and TIMP-2) capable to activate in a cooperative manner on cell surface pro-MMP-2 [10, 20] further support this hypothesis.

## 5. Conclusions

We demonstrate that fibroblasts, derived from MDE, produce low levels of TIMP-1, the major inhibitor of MMPs, and elevated levels of MMP-2. MMP-14 and TIMP-2 capable to activate in a cooperative manner pro-MMP-2 are also present in MDE areas. These data suggest that cooperation of different cell populations (i.e., inflammatory cells and fibroblasts) may result in an increased elastolytic burden that is caused of a focal loss of elastic tissue in the midreticular dermis of MDE patients.

## References

[1] Shelley W. B., Wood M. G. (1977). Wrinkles due to idiopathic loss of mid-dermal elastic tissue. *British Journal of Dermatology*.

[2] Patroi I., Annessi G., Girolomoni G. (2003). Mid-dermal elastolysis: a clinical, histologic, and immunohistochemical study of 11 patients. *Journal of the American Academy of Dermatology*.

[3] Gambichler T. (2010). Mid-dermal elastolysis revisited. *Archives of Dermatological Research*.

[4] Fimiani M., Mazzatenta C., Alessandrini C., Marcolongo P., Calzoni P., Andreassi L. (1995). Mid-dermal elastolysis: an ultrastructural and biochemical study. *Archives of Dermatological Research*.

[5] Cavarra E., Fimiani M., Lungarella G. (2002). UVA light stimulates the production of cathepsin G and elastase-like enzymes by dermal fibroblasts: a possible contribution to the remodeling of elastotic areas in sun-damaged skin. *Biological Chemistry*.

[6] Gambichler T., Skrygan M. (2013). Decreased lysyl oxidase-like 2 expression in mid-dermal elastolysis. *Archives of Dermatological Research*.

[7] Martinez-Escala M. E., Rozas E., Pujol R. M., Herrero-Gonzalez J. E. (2012). Mid-dermal elastolysis: another dermatological clue to autoimmunity?. *Acta Dermato-Venereologica*.

[8] Wagner G., Sachse M. M. (2011). Elastolysis mediodermalis - case report and review of literature. *Journal der Deutschen Dermatologischen Gesellschaft*.

[9] Lewis K. G., Berkovitch L., Dill S. W., Robinson-Bostom L. (2004). Acquired disorders of elastic tissue: part II. Decreased elastic tissue. *Journal of the American Academy of Dermatology*.

[10] Sariahmetoglu M., Crawford B. D., Leon H. (2007). Regulation of matrix metalloproteinase-2 (MMP-2) activity by phosphorylation. *FASEB Journal*.

[11] Ortel B., Rappersberger K., Konrad K. (1992). Middermal elastolysis in an elderly man with evidence of elastic fiber phagocytosis. *Archives of Dermatology*.

[12] Sterling J. C., Coleman N., Pye R. J. (1994). Mid-dermal elastolysis. *British Journal of Dermatology*.

[13] Neri I., Patrizi A., Fanti P. A., Passarini B., Badiali-De Giorgi L., Varotti C. (1996). Mid-dermal elastolysis: a pathological and ultrastructural study of five cases. *Journal of Cutaneous Pathology*.

[14] Lungarella G., Cavarra E., Lucattelli M., Martorana P. A. (2008). The dual role of neutrophil elastase in lung destruction and repair. *The International Journal of Biochemistry & Cell Biology*.

[15] Mechams R. P., Broekelmann T. J., Fliszar C. J., Shapiro S. D. (1997). Elastin degradation by matrix metalloproteinases. *The Journal of Biological Chemistry*.

[16] Lungarella G., Cavarra E., Fineschi S., Lucattelli M., Vergnolle N., Chignard M. (2011). Dual role for proteases in lung inflammation. *Proteases and their Receptors in Inflammation, Progress in Inflammation Research*.

[17] Sallenave J.-M., Shapiro S. D. (2008). Proteases and antiproteases in development, homeostasis and disease: the old, the new, and the unknown. *The International Journal of Biochemistry & Cell Biology*.

[18] Löffek S., Schilling O., Franzke C.-W. (2011). Biological role of matrix metalloproteinases: a critical balance. *European Respiratory Journal*.

[19] Manicone A. M., McGuire J. K. (2008). Matrix metalloproteinases as modulators of inflammation. *Seminars in Cell & Developmental Biology*.

[20] Ra H.-J., Parks W. C. (2007). Control of matrix metalloproteinase catalytic activity. *Matrix Biology*.

